# Candida albicans Promotes Oral Cancer via IL-17A/IL-17RA-Macrophage Axis

**DOI:** 10.1128/mbio.00447-23

**Published:** 2023-04-17

**Authors:** Xu Wang, Shuangshaung Wu, Wenjie Wu, Wenqing Zhang, Linman Li, Qian Liu, Zhimin Yan

**Affiliations:** a Department of Oral Medicine, Peking University School and Hospital of Stomatology & National Center for Stomatology & National Clinical Research Center for Oral Diseases & National Engineering Research Center of Oral Biomaterials and Digital Medical Devices, Beijing, People’s Republic of China; b Department of Oral and Maxillofacial Surgery, Peking University School and Hospital of Stomatology & National Center for Stomatology & National Clinical Research Center for Oral Diseases & National Engineering Research Center of Oral Biomaterials and Digital Medical Devices, Beijing, People’s Republic of China; c Central Laboratory, Peking University School and Hospital of Stomatology, Beijing, People’s Republic of China; McMaster University

**Keywords:** *Candida albicans*, oral cancer, IL-17A, macrophage, tumor microenvironment

## Abstract

The association between Candida albicans (C. albicans) and oral cancer (OC) has been noticed for a long time, but the mechanisms for C. albicans promoting OC are rarely explored. In this study, we determined that C. albicans infection promoted OC incidence in a 4-nitroquinoline 1-oxide (4NQO)-induced mouse tongue carcinogenesis model as well as promoted OC progression in a tongue tumor-bearing mouse model (C3H/HeN-SCC VII). We then demonstrated that tumor-associated macrophage (TAMs) infiltration was elevated during C. albicans infection. Meanwhile, the attracted TAMs polarized into M2-like macrophages with high expression of programmed death ligand 1 (PD-L1) and galectin-9 (GAL-9). Further analysis suggested that the interleukin (IL)-17A/IL-17RA pathway activated in OC cells was a contributor to the excessive TAMs infiltration in C. albicans-infected mice. Thus, we constructed IL-17A neutralization and macrophage depletion experiments in C3H/HeN-SCC VII mice to explore the role of IL-17A/IL-17RA and TAMs in OC development caused by C. albicans infection. The results showed that both IL-17A neutralization and macrophage depletion tended to reduce the TAMs number and tumor size in mice with C. albicans infection. Collectively, our finding revealed that C. albicans promoted OC development via the IL-17A/IL-17RA-macrophage axis, opening perspectives for revealing C. albicans-tumor immune microenvironment links.

## INTRODUCTION

A growing body of research has reported microbiomes’ role in the diagnosis, development, progression, and treatment response of multiple cancers (1, 2). Not surprisingly, the polymorphic microbiome was suggested to be one of the hallmarks of cancer (3). However, most of the attention has been focused on bacteria and viruses, and relatively few studies have explored the role of fungi in cancer (4, 5).

Undoubtedly, fungi are important players during cancer development. It has been demonstrated that tumor fungal richness varied significantly across cancer types (5). Meanwhile, an association between transcriptionally active *Candida* and gastrointestinal cancers including oral, stomach, and colon cancers has been recognized (4). In particular, among the limited evidence concerning fungi-cancer links, the association between Candida albicans (C. albicans) and oral cancer (OC) has been noticed by dentists for many years, which may be a typical example of exploring the fungi-cancer links (6).

OC is the malignant neoplasm of the lip and oral cavity according to the International Classification of Diseases (ICD-10). It has been estimated that 377,713 new cases and 177,757 deaths of OC worldwide in 2020 (7). Tobacco smoking, betel quid chewing, alcohol consumption, and human papillomavirus (HPV) infection are accepted risk factors for OC (8). Now, increasing evidence has revealed that microorganisms such as *Fusobacteria* and C. albicans may be potential carcinogenic factors for OC (9).

C. albicans is the most common fungus inhabiting the human oral cavity. The association between C. albicans, chronic hyperplastic candidosis (CHC), and OC was recognized around the 1960s (10, 11). Since then, increasing studies reported the malignant transformation of CHC (12). In addition, some clinical studies suggested that oral *Candida* presence might be a risk factor for OC (13, 14). However, it is still a debate about whether C. albicans infection is a contributor to OC development (15). Several animal studies may provide clues about the role of C. albicans infection in promoting OC development (16–20). In particular, recent studies demonstrated that C. albicans increased OC incidence in a 4-nitroquinoline 1-oxide (4NQO)-induced mouse tongue carcinogenesis model (16) and promoted OC progression in a xenograft mouse model (17). Nevertheless, the underlying mechanisms for C. albicans promoting OC are complex and still undetermined.

Some hypotheses have been proposed to elucidate the mechanisms for C. albicans promoting OC, such as the production of carcinogenic by-products, triggering of inflammation, induction of T-helper 17 (Th17) response, and molecular mimicry (21). Among them, the production of nitrosamines (22) and acetaldehyde (23) may be the most discussed. Additionally, Vadovics et al. (17) found that C. albicans might promote OC progression by inducing inflammatory and metastatic gene expression and epithelial-mesenchymal transition. Beyond these, it makes sense to reveal whether C. albicans can promote OC by influencing the tumor immune microenvironment (TIME).

Considering the close association between C. albicans and OC, and the unrevealed map of the C. albicans-TIME-tumor axis, this study further demonstrated the role of C. albicans in promoting OC development and revealed the underlying mechanisms from the perspective of TIME by *in vivo* and *in vitro* experiments.

## RESULTS

### C. albicans infection increased oral cancer incidence in 4NQO mice.

**FIG 1:**
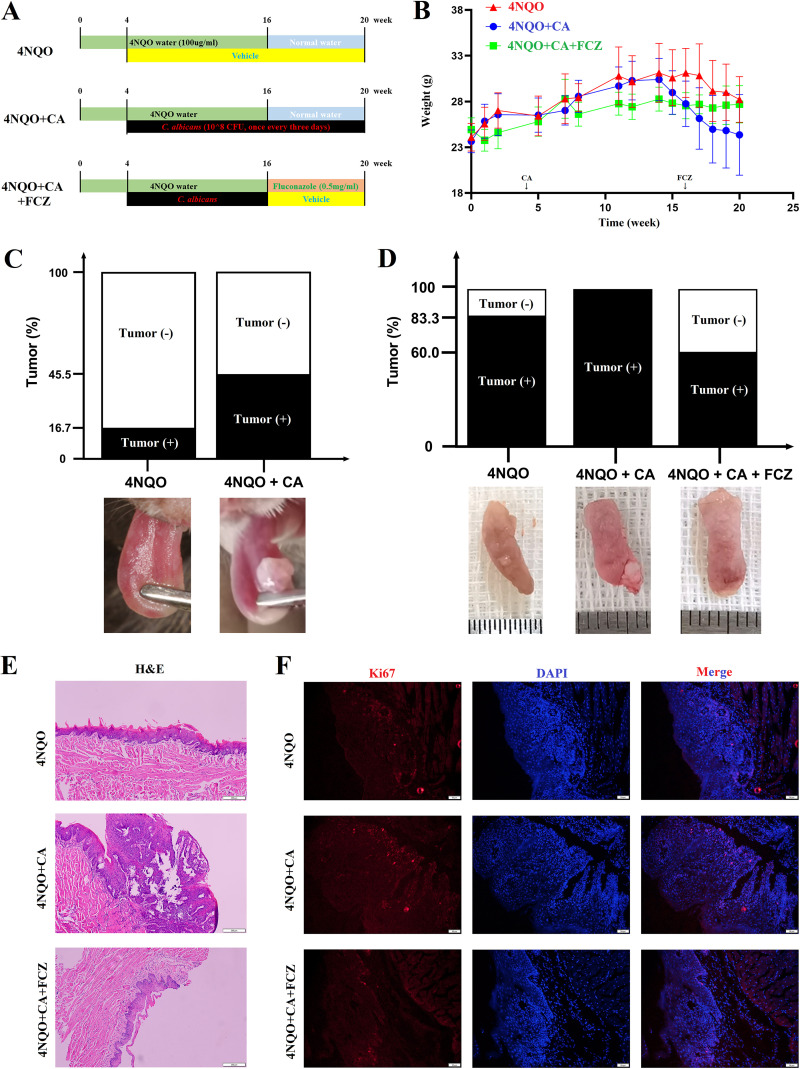
C. albicans infection promoted oral cancer incidence in 4NQO mice model. (A) Schematic figure of 4NQO mice with C. albicans infection. (B) Body weight of 4NQO mice during the experiment. (C) Tongue tumor incidence in week 16; the bottom is the representative tongue images. (D) Tongue tumor incidence in week 20; the bottom is the representative tongue images. (E) Representative H&E images of the tongue mucosa; scale bar = 200 μm. (F) Representative IF images of Ki-67 (red) expression in the tongue mucosa; scale bar = 50 μm.

During the experiment (Fig. 1A), C. albicans infection caused weight loss in the 4NQO mice, which was reversed by the administration of fluconazole (FCZ) (Fig. 1B). In week 16, the incidence of oral tumor in the 4NQO + CA group was approximately three times higher than in the 4NQO group (45.5% versus 16.7%, Fig. 1C and Fig. S1A). In week 20, the tumor incidence in the 4NQO + CA group reached 100%, which was still higher than the 4NQO group (83.3%) (Fig. 1D and Fig. S1B). Interestingly, the tumor incidence in the 4NQO + CA + FCZ group was lower than both the 4NQO + CA and 4NQO groups (Fig. 1D and E and Fig. S1B), which suggested that antifungal treatment effectively reversed the tumor development caused by C. albicans infection. Additionally, the Ki-67 (a proliferation marker) expression of tongue epithelium in the 4NQO + CA group was higher than the 4NQO group and the 4NQO + CA + FCZ group (Fig. 1F), which also means the higher risk of tumorigenesis in mice with C. albicans infection.

10.1128/mbio.00447-23.1 FIG S1 C. albicans infection promoted oral tumor incidence and changed peripheral immune cells in 4NQO mice. (A) Tongue figures of the mice in 16 weeks; the white circles represent the tumors; one mouse in 4NQO + CA group died of nontumor cause. (B) Tongue figures of the mice in 20 weeks; the white circles represent the tumors; the black box in 4NQO + CA group means the dead mice because of oral cancer. (C and D) Representative pseudocolor figures (C) and their statistical results (D) of the peripheral immune cells (CD4^+^T cells, CD8^+^ T cells, DC cells, B cells, macrophages, MDSCs, and NK cells) in 4NQO mice. ***, *P* < 0.001; ****, *P* < 0.0001; ns, not significant. Download FIG S1, TIF file, 7.5 MB. Copyright © 2023 Wang et al. 2023 Wang et al. https://creativecommons.org/licenses/by/4.0/ This content is distributed under the terms of the Creative Commons Attribution 4.0 International license.

Moreover, there were differences in the immune cell subtypes of peripheral blood between groups. When compared with the 4NQO group, the CD3^+^CD4^+^ T cells and CD3^−^CD19^+^ B cells were downregulated, but the CD3^+^CD8^+^ T cells, CD11b^+^Gr-1^+^ myeloid-derived suppressor cells (MDSCs), and CD11b^+^F4/80^+^ macrophages were upregulated in the 4NQO + CA group (Fig. S1C and D).

### Tumor-associated macrophage accumulation was associated with IL-17A/IL-17RA signaling pathway during C. albicans infection.

RNA sequencing (RNA-seq) was used to screen the possible mechanisms for C. albicans promoting OC. One hundred twenty-eight differentially expressed genes (DEGs) were filtered between the 4NQO and 4NQO + CA group (Fig. S2A), 239 DEGs were found between the 4NQO + CA and 4NQO + CA + FCZ group (Fig. S2B), and only 27 DEGs were found between the 4NQO and 4NQO + CA + FCZ group (Fig. S2C). The administration of FCZ reversed some of the changed genes in tumors caused by C. albicans infection (Fig. S2D and E).

10.1128/mbio.00447-23.2 FIG S2 C. albicans infection changed the gene expression in tumors from 4NQO mice. (A to C) The differentially expressed genes between 4NOQ and 4NQO + CA group (A), 4NOQ + CA and 4NQO + CA + FCZ group (B), as well as 4NOQ and 4NQO + CA + FCZ group (C). (D) The downregulated genes in CA group (A < O) revised by FCZ administration (F > A). (E) The upregulated genes in CA group (A > O) revised by FCZ administration (F < A). (F) RT-qPCR results of the *S100A8*, *MMP-3*, and *CXCL10* mRNA levels in the tumors from 4NQO mice. (G) Gene set enrichment analysis (GSEA) result of IL-17 signaling pathway in tongue epithelium from normal mice with eight-month C. albicans infection (CA) versus without infection (BLK); transcriptome analysis was performed with the tongue epithelium from both groups (sequence data that support this finding has been deposited in BioProject with the primary accession code PRJNA944155 and in SRA with the primary accession code SRP427175). (H and I) GSEA results of IL-17 signaling pathway in tongue epithelium or tumor from mice with 4NQO water drinking for 24 weeks versus week 0 (H) and week 12 versus week 0 (I), performed with publicly available RNA-seq dataset (GSE101469). (J) Correlations between S100A8, MMP-3, CXCL10 FPKM, and macrophage infiltration level based on the mRNA-seq data from 4NQO mice. *, *P* < 0.05; **, *P* < 0.01; ns, not significant. Download FIG S2, TIF file, 6.1 MB. Copyright © 2023 Wang et al. 2023 Wang et al. https://creativecommons.org/licenses/by/4.0/ This content is distributed under the terms of the Creative Commons Attribution 4.0 International license.

**FIG 2:**
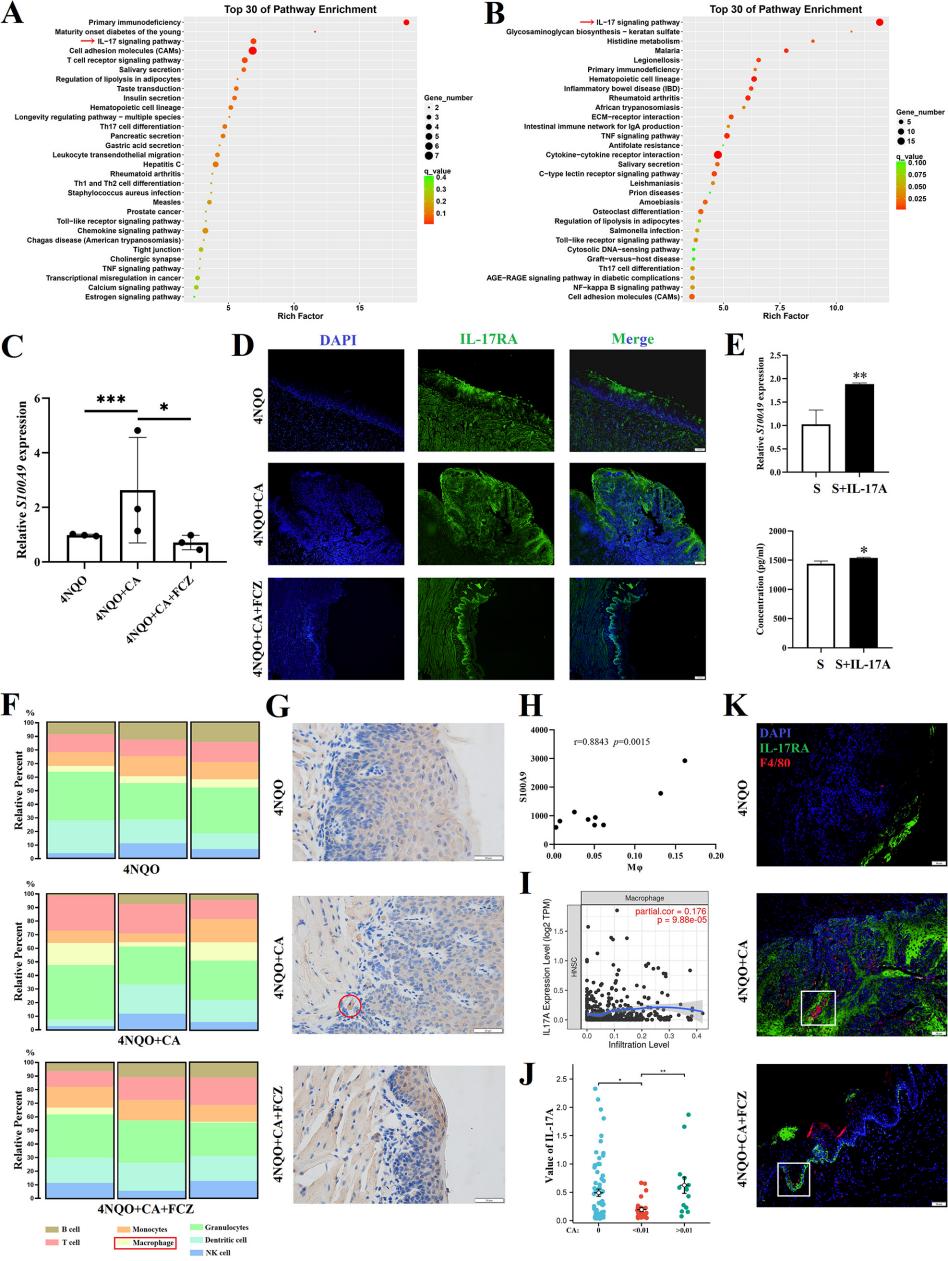
Macrophage infiltration was correlated with IL-17A/IL-17RA signaling pathway. (A and B) KEGG enrichment analysis showed the top 30 enrichment pathways between 4NQO and 4NQO + CA group (A) and 4NQO + CA and 4NQO + CA + FCZ group (B). (C) RT-qPCR results of the S100A9 mRNA levels in tumors from 4NQO mice. (D) Representative IF images of IL-17RA (green) expression in the tongue mucosa; scale bar = 100 μm. (E) S100A9 expression in SCC VII cells; the top represents the mRNA expression levels; the bottom represents the released protein levels measured by ELISA; S (SCC VII cells), S + IL-17A (SCC VII cells treated with 100 ng/mL rmIL-17A). (F) Tumor immune cell infiltration based on the mRNA-seq data (calculated by ImmuCellAI-mouse (http://bioinfo.life.hust.edu.cn/ImmuCellAI-mouse/#!)). (G) Representative IHC images of F4/80 expression in the tongue epithelium; red circle represents the F4/80^+^ macrophage; scale bar = 50 μm. (H) Correlation between S100A9 FPKM and macrophage infiltration level based on the mRNA-seq data. (I) Correlation between IL-17A expression level and macrophage infiltration level in HNSC from the TCGA data in TIMER (https://cistrome.shinyapps.io/timer/). (J) Association between IL-17A level and C. albicans detected in HNSC from the TCGA data. (K) Representative IF images of IL-17RA (green) and F4/80 (red) expression in the tongue mucosa; white square indicates that more macrophages surrounded the epithelial cells and tumor cells with higher IL-17RA expression; scale bar = 50 μm. *, *P* < 0.05; **, *P* < 0.01; ***, *P* < 0.001.

Among the DEGs, the interleukin (IL)-17 signaling pathway was noticeable from the Kyoto Encyclopedia Genes and Genomes (KEGG) enrichment analysis, which was upregulated in 4NQO + CA mice (Fig. 2A) and downregulated after FCZ treatment (Fig. 2B). Meanwhile, the upregulation of downstream molecules (*S100A9*, *S100A8*, *MMP3*, and *CXCL10*) of IL-17RA activation in the 4NQO + CA mice was confirmed by quantitative real-time PCR (RT-qPCR) (Fig. 2C and Fig. S2F). To identify the IL-17RA expression cells, immunofluorescence (IF) was performed and found that epithelial cells and tumor cells were the main cells expressing IL-17RA (Fig. 2D). Thus, an *in vitro* experiment was designed and showed that SCC VII cells expressed more S100A9 when treated with rmIL-17A (Fig. 2E), which confirmed activation of the IL-17A/IL-17RA signaling pathway in oral tumor cells.

It is worth mentioning that our ongoing work found that there was no difference in the IL-17 signaling pathway of the tongue epithelium between long-term (8 month) C. albicans-infected mice and normal mice (Fig. S2G). Additionally, a publicly available RNA-seq data set (GSE101469) (24) was used to find that the IL-17 signaling pathway was significantly upregulated in carcinoma (C57BL/6 mice drank 50 mg/L 4NQO water for 28 weeks) compared with normal lingual mucosa (4NQO for week 0) (Fig. S2H), while there was no significant difference between 4NQO for weeks 0 and 12 (hyperplasia and mild and moderate dysplasia) (Fig. S2I). The above results may indicate an activation of the IL-17A/IL-17RA signaling pathway in the tumor microenvironment (TME) during oral cancer development, rather than just the mucosal immune response caused by C. albicans infection.

Furthermore, the tumor immune cell infiltration was estimated from the RNA-seq data set by the use of ImmuCellAI-mouse (http://bioinfo.life.hust.edu.cn/ImmuCellAI-mouse/#!). The results showed that the macrophage infiltration level was upregulated in the 4NQO + CA group, compared with the 4NQO group and the 4NQO + CA + FCZ group (Fig. 2F), which was validated by immunohistochemistry (IHC) of F4/80 (a mouse macrophage marker) (Fig. 2G).

Interestingly, a possible relationship was noticed between IL-17A/IL-17RA activation and macrophage infiltration. Thus, correlation analysis was performed and confirmed the significant positive correlations between IL-17A/IL-17RA downstream molecule (S100A9, S100A8, MMP3, and CXCL10) levels and macrophage infiltration levels (Fig. 2H and Fig. S2J).

In addition, a positive correlation was also found between IL-17A expression level and macrophage infiltration level in head and neck squamous cell carcinoma (HNSC) (Fig. 2I) from the TCGA data in TIMER web server (https://cistrome.shinyapps.io/timer/). Meanwhile, the association between C. albicans detected in HNSC (4) and IL-17A in matched samples from TCGA data was analyzed. When the samples were divided into undetected (C. albicans = 0, *n* = 72), low abundance (0 ~ 0.01, *n* = 24), and high abundance (>0.01, *n* = 14) groups according to the abundance of C. albicans, it was found that IL-17A expression in the high-abundance group was higher than low-abundance group (*P* < 0.05) and undetected group (*P* > 0.05) (Fig. 2J and Table S1).

10.1128/mbio.00447-23.6 TABLE S1 Data used for analyzing the association between C. albicans detected in HNSC and IL-17A in matched samples from the TCGA database. Download Table S1, XLSX file, 0.02 MB. Copyright © 2023 Wang et al. 2023 Wang et al. https://creativecommons.org/licenses/by/4.0/ This content is distributed under the terms of the Creative Commons Attribution 4.0 International license.

It should be noted that macrophage level was estimated from the RNA-seq data, the calculation of which may include the expression of IL-17A, IL-17RA, and their downstream molecules. Thus, IF was performed and showed that more macrophages surrounded the epithelial cells and tumor cells with higher IL-17RA expression (Fig. 2K). Thus, excessive infection of C. albicans may cause more IL-17A production and release, which further activates the IL-17A/IL-17RA signaling pathway in OC cells to induce macrophage infiltration.

### Oral tumor cells treated with IL-17A attracted macrophages and induced macrophages into immunosuppressive phenotype.

**FIG 3:**
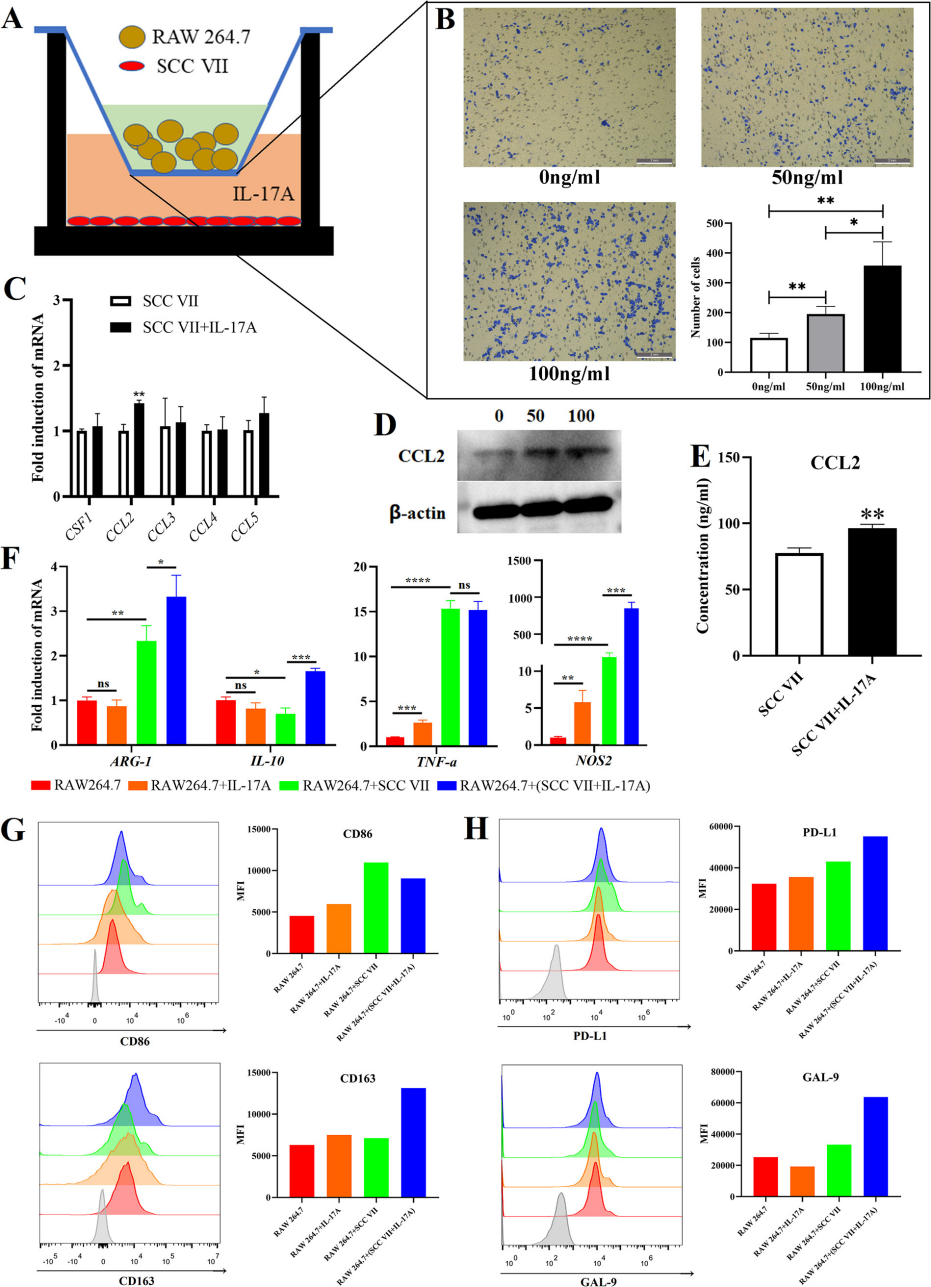
Oral cancer cells with IL-17RA activation attracted macrophages and modified macrophages into immunosuppressive phenotype. (A) Schematic figure of Transwell migration experiment. (B) Results of Transwell migration experiment. (C) mRNA expression levels of chemokines associated with macrophage attraction in SCC VII cells; IL-17A with 100 ng/mL. (D) WB of CCL2 in SCC VII cells; IL-17A with 0, 50, or 100 ng/mL. (E) ELISA results of CCL2 released by SCC VII; IL-17A with 100 ng/mL. (F) mRNA levels of M1- and M2-like macrophage related genes in RAW 264.7 cells; IL-17A with 100 ng/mL. (G) FCM results of CD86 and CD163 on RAW 264.7 cells; IL-17A with 100 ng/mL. (H) FCM results of PD-L1 and GAL-9 on RAW 264.7 cells; IL-17A with 100 ng/mL. *, *P* < 0.05; **, *P* < 0.01; ***, *P* < 0.001; ****, *P* < 0.0001; ns, not significant.

Based on the above relationship between IL-17A/IL-17RA signaling activation and macrophage infiltration in oral tumors, a Transwell migration experiment was designed (Fig. 3A) and found that SCC VII cells treated with rmIL-17A attracted more RAW 264.7 cells into the lower chamber (Fig. 3B). Then, among the chemokines associated with macrophage attraction, the chemokine (C-C motif) ligand 2 (*CCL2*) gene level in SCC VII cells (Fig. 3C) and CCL2 protein concentration in cell extract (Fig. 3D) and cultivate supernatant (Fig. 3E) were upregulated when SCC VII cells were treated with rmIL-17A. Thus, the OC cells with IL-17RA activation released more CCL2 to attract macrophages into the tumor environment.

Furthermore, the influence of OC cells with IL-17RA activation on the phenotype of macrophages was explored. When RAW 264.7 cells were directly treated with rmIL-17, the M1-like markers (*NOS2* and *TNF-α*) were upregulated, but M2-like markers (*ARG-1* and *IL-10*) were not influenced (Fig. 3F). When RAW 264.7 cells were cultured with supernatant from SCC VII cells, both M1- and M2-like markers (*ARG-1*, *NOS2,* and *TNF-α*) were upregulated, with M1-like markers being dominant (Fig. 3F). Interestingly, the supernatant from SCC VII cells pretreated with rmIL-17A caused more significant upregulation of M2-like markers (*ARG-1* and *IL-10*) in macrophages compared with supernatant from SCC VII cells alone (Fig. 3F). With flow cytometry (FCM), a similar trend could be found in the expression of CD86 (M1) and CD163 (M2) on the macrophages (Fig. 3G). The above results indicated that the separate existence of IL-17A or OC cells induced a polarization dominated by M1-like macrophages, but the OC cells pretreated with IL-17A could promote M2 polarization.

Additionally, the coinhibitory factors (programmed death ligand 1 [PD-L1] and galectin-9 [GAL-9]) of the immune response were also upregulated on RAW 264.7 cells treated with cultivated supernatant from SCC VII + rmIL-17A (Fig. 3H). Moreover, the RAW 264.7 cells directly treated with C. albicans also showed an upregulation of *NOS2*, *IL-10*, *CD80*, *CD86*, *CD274*, and *LGALS9* (Fig. S3A and B).

10.1128/mbio.00447-23.3 FIG S3 C. albicans infection induced the tumor-associated macrophages into an immunosuppressive phenotype. (A) C. albicans treatment induced *NOS2* and *IL-10* upregulation in RAW 264.7 cells. (B) C. albicans treatment upregulated coinhibitory factors (*CD274*, *LGALS9*) in RAW 264.7 cells. (C) C. albicans infection upregulated CCL2 in tumors from the C3H/HeN-SCC VII mice; the top represents WB result; the bottom represents ELISA result. (D) C. albicans infection upregulated CD163, PD-L1, and GAL-9 on TAMs in C3H/HeN-SCC VII mice.*, *P* < 0.05; **, *P* < 0.01; ***, *P* < 0.001; ****, *P* < 0.0001; ns, not significant. Download FIG S3, TIF file, 1.1 MB. Copyright © 2023 Wang et al. 2023 Wang et al. https://creativecommons.org/licenses/by/4.0/ This content is distributed under the terms of the Creative Commons Attribution 4.0 International license.

*In vivo* experiments further demonstrated the above *in vitro* results. C. albicans infection caused the upregulation of CCL2 (Fig. S3C) concentration as well as CD163, PD-L1, and GAL-9 expression on tumor-associated macrophages (TAMs) (Fig. S3D) in oral tumors isolated from C3H/HeN-SCC VII mice.

### The neutralization of IL-17A alleviated the tumor progression promoted by C. albicans infection.

**FIG 4:**
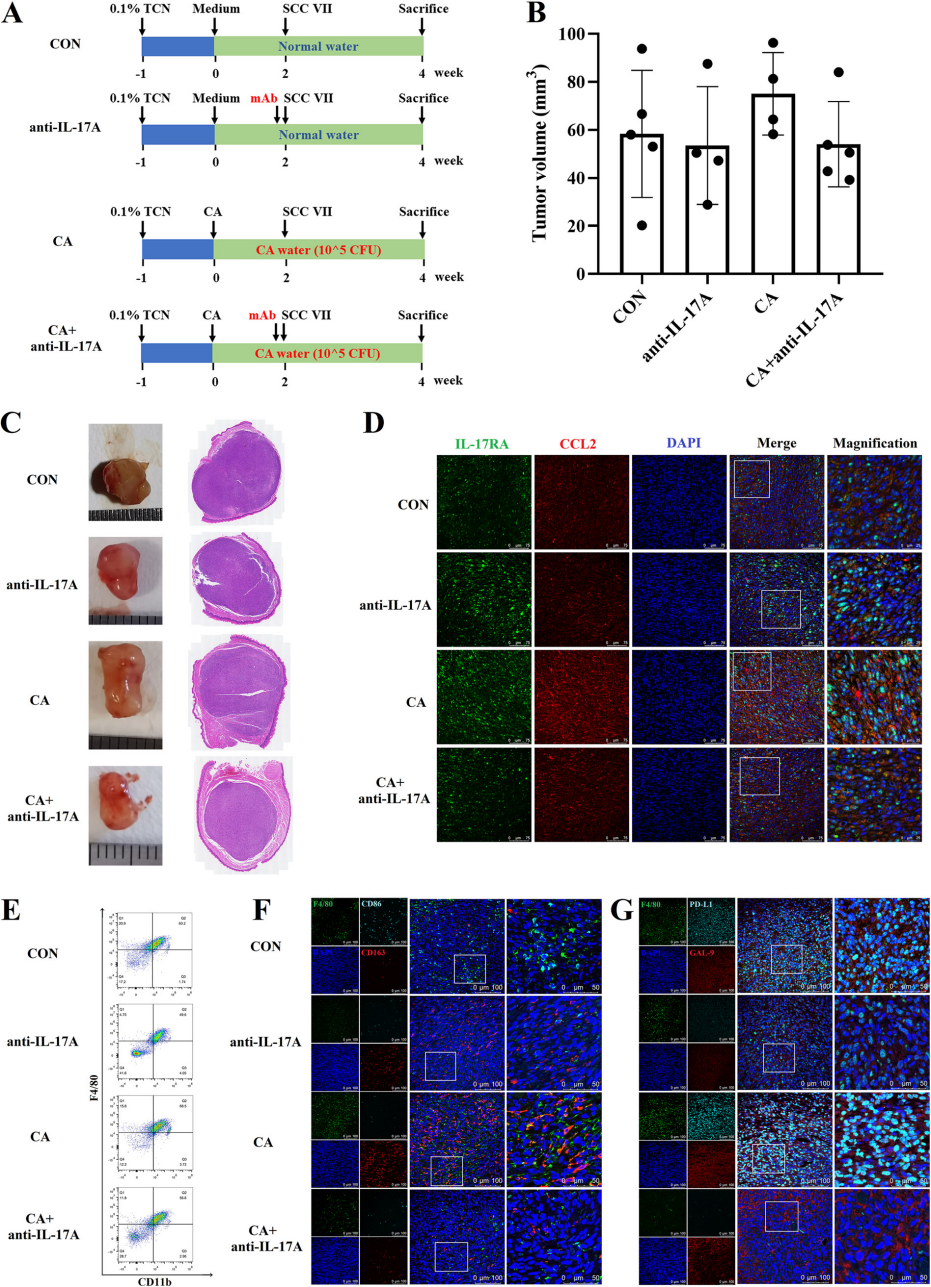
The neutralization of IL-17A alleviated the oral tumor progression promoted by C. albicans infection. (A) Schematic figure of IL-17A neutralization experiment in C3H/HeN-SCC VII mice; CON (C3H/HeN-SCC VII), anti-IL-17A (C3H/HeN-SCC VII with IL-17A neutralization), CA (C3H/HeN-SCC VII with C. albicans infection), and CA + anti-IL-17A (C3H/HeN-SCC VII with C. albicans infection and IL-17A neutralization). (B) Tumor volume. (C) Representative tumor images and H&E images. (D) Representative IF images of IL-17RA (green) and CCL2 (red); scale bar = 75 μm or 25 μm. (E) FCM results of CD11b^+^F4/80^+^ macrophages. (F) Representative IF images of F4/80 (green), CD86 (indigo blue), and CD163 (red); scale bar = 100 μm or 50 μm. (G) Representative IF images of F4/80 (green), PD-L1 (indigo blue), and GAL-9 (red); scale bar = 100 μm or 50 μm.

An *in vivo* experiment was designed to confirm the role of IL-17A in C. albicans promoting OC (Fig. 4A). Although there was no statistical difference, the oral tumor in the CA group was bigger than the CON group (*P* = 0.3144) and was reduced after the neutralization of IL-17A (*P* = 0.117*3*) (Fig. 4B and C and Fig. S4A). The CCL2 upregulation caused by C. albicans infection was downregulated after the neutralization of IL-17A (Fig. 4D), which might further result in decreased TAMs infiltration (Fig. 4E and Fig. S4B).

10.1128/mbio.00447-23.4 FIG S4 The neutralization of local IL-17A alleviated the tumor progression promoted by C. albicans infection. (A) Tumors isolated from the tongue of C3H/HeN-SCC VII mice; one mouse in anti-IL-17A group died of nontumor cause; the black box in CA group means the dead mice because of oral cancer. (B) Local IL-17A neutralization reduced the macrophages attracted to tumors. **, *P* < 0.01; ns, not significant. Download FIG S4, TIF file, 1.9 MB. Copyright © 2023 Wang et al. 2023 Wang et al. https://creativecommons.org/licenses/by/4.0/ This content is distributed under the terms of the Creative Commons Attribution 4.0 International license.

Additionally, more M2-like macrophages (CD163^+^) accumulated into the tumor from mice with C. albicans infection and reduced after IL-17A neutralization (Fig. 4F). The PD-L1 expression on macrophages was also upregulated in the CA group than CON group and seemed to be reversed after the neutralization of IL-17A (Fig. 4G).

### The depletion of macrophages alleviated the tumor progression promoted by C. albicans infection.

**FIG 5:**
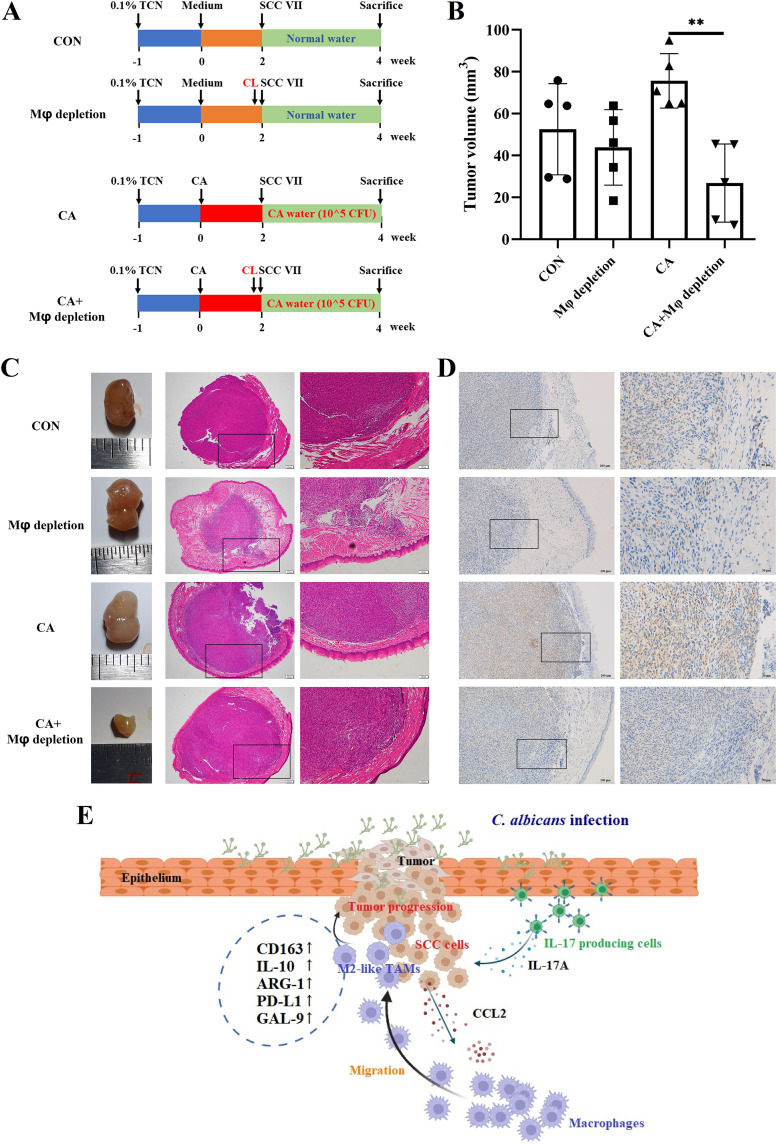
The depletion of local macrophages alleviated the oral tumor progression promoted by C. albicans infection. (A) Schematic figure of local macrophage depletion experiment in C3H/HeN-SCC VII mice; CON (C3H/HeN-SCC VII), Mφ depletion (C3H/HeN-SCC VII with macrophage depletion), CA (C3H/HeN-SCC VII with C. albicans infection), and CA + Mφ depletion (C3H/HeN-SCC VII with C. albicans infection and macrophage depletion). (B) Tumor volume. (C) Representative tumor images and H&E images, scale bar = 200 μm or 100 μm. (D) Representative IHC images of F4/80; scale bar = 200 μm or 50 μm. (E) Mechanism diagram of this research (drawing with online software, https://app.biorender.com/, agreement number is FQ256A5VYW). C. albicans infection promoted IL-17A production and the following IL-17RA signal activation in oral tumor cells; then, tumor cells released CCL2 to attract macrophages into TME; the macrophages in TME showed an immunosuppressive phenotype with elevated expressions of PD-L1, GAL-9, and M2-like macrophage markers. **, *P* < 0.01.

Furthermore, the role of macrophages in C. albicans promoting OC was confirmed *in vivo* (Fig. 5A). When without C. albicans infection, there was no significant difference in tumor size between Mφ depletion and CON group (Fig. 5B and C and Fig. S5A). However, when accompanied by C. albicans infection, the depletion of local macrophages significantly reduced the tumor size (*p* = 0.0013) (Fig. 5B and C and Fig. S5A). The depletion of tumor-infiltrated macrophages was validated by IHC (Fig. 5D). The above results indicated that the decreased tumor volume in the CA + Mφ depletion group may be an effect of C. albicans infection and macrophage depletion together rather than macrophage depletion alone.

10.1128/mbio.00447-23.5 FIG S5 The depletion of macrophages alleviated the oral tumor progression promoted by C. albicans infection. (A) Tumors isolated from the tongue of C3H/HeN-SCC VII mice. Download FIG S5, TIF file, 5.5 MB. Copyright © 2023 Wang et al. 2023 Wang et al. https://creativecommons.org/licenses/by/4.0/ This content is distributed under the terms of the Creative Commons Attribution 4.0 International license.

## DISCUSSION

The association between C. albicans infection and OC development has been recognized for decades, but the mechanisms for C. albicans promoting OC still need to be explored. This study confirmed the role of C. albicans in promoting OC and further systematically explored the underlying mechanisms from the perspective of TIME. Specifically, the infection of C. albicans caused the activation of the IL-17A/IL-17RA signaling pathway in OC cells. Then, the activated OC cells released more CCL2 to attract macrophages. Meanwhile, the attracted macrophages changed into an immunosuppressive phenotype in the TME. As a result, an immunosuppressive microenvironment facilitating OC development was shaped with the participation of C. albicans, IL-17A, and TAMs (Fig. 5E).

IL-17/IL-17R, especially IL-17A/IL-17RA, is an important pathway for the host against extracellular bacteria and fungi (25). Nonhematopoietic cells such as oral epithelial cells, skin keratinocytes, fibroblasts, and gut epithelial cells are the main responders to IL-17, which are the providers of inflammatory cytokines (IL-6 and IL-1), chemokines (CXCL2, CCL2, and CCL20), antimicrobial peptides (β-defensins and S100 proteins), and matrix metalloproteinases (MMP1, MMP3, and MMP9) during IL-17RA activation (25). However, the unrestrained IL-17 signal is proposed to be associated with cancer progression (26).

Elevated IL-17 signature genes can be found in multiple human cancers such as cervical cancer, esophageal cancer, gastric cancer, hepatocellular carcinoma, and colorectal cancer (26). It is interesting to note that mucosal sites with microbial colonization seem to be the main victims of the above cancers with IL-17A/IL-17RA hyperactivation. Coincidentally, the relationships between C. albicans infection and esophageal cancer (22), gastric cancer (27), hepatocellular carcinoma (28), and colorectal cancer (29) have been revealed. Thus, it is speculated that microbial dysbiosis including the overgrowth of C. albicans may be an important contributor to the unrestrained IL-17 signaling pathway and the subsequent cancer progression.

In the present study, IL-17A signature gene levels were correlated with OC progression in mice with C. albicans infection. Moreover, the neutralization of IL-17A partly alleviated the cancer progression caused by C. albicans infection. Some studies have also revealed that bacteria might promote the progression of colon cancer and gastric cancer by IL-17 induction (30, 31). This study further demonstrated the role of IL-17 in OC progression caused by C. albicans infection, which adds evidence for the fungi/bacteria-IL-17-cancer axis.

Th17 cells, Tc17 cells (IL-17-producing CD8^+^ T cells), and some innate immune subsets such as γδ-T cells, natural killer T (NKT) cells, and type 3 “innate lymphoid cells” (ILC3) can express IL-17 (25). Among them, IL-17-producing γδT cells have been shown to promote breast cancer metastasis (32) and colorectal cancer development (33). Our further work will determine which IL-17-producing cell types are the key actors for C. albicans promoting OC development.

TAMs are also double-edged swords in TME with the ability to promote cancer or inhibit cancer (34). TAMs have been shown to promote tumor cell survival and proliferation, angiogenesis, and suppression of immune responses (34, 35). Our data showed excessive TAM infiltration in mice with C. albicans infection. Through *in vitro* experiments, we found an upregulation of CCL2 in OC cells treated with IL-17A and the subsequent attraction of macrophages. Meanwhile, the *in vivo* experiment demonstrated the reduction of TAMs after local IL-17A neutralization. Furthermore, the depletion of macrophages alleviated the tumor progression promoted by C. albicans infection. Thus, IL-17A might promote OC by the attraction of macrophages.

A recent single-cell-RNA-sequencing study reported that certain clusters of macrophages might participate in OC carcinogenesis with C. albicans infection (36). However, it is still undetermined which clusters of TAMs participate in this process. Our data showed that C. albicans infection induced M2-like TAMs with PD-L1 and GAL-9 upregulation in TME. Both OC cells with IL-17RA activation and C. albicans are contributors to this kind of TAMs. It is noteworthy that M1- and M2-macrophages *in vitro* are not representative of their *in vivo* states, and their associated gene expression is widely distributed with overlapping populations and heterogeneous patterns (37). With recent progress in genomics, single-cell-RNA-sequencing, and time-of-flight technologies, macrophages can be classified into more distinct clusters beyond M1- and M2-polarized phenotypes (38). Thus, more specific TAM phenotypes in C. albicans promoting OC need to be identified.

The polarization mechanisms of macrophages are complex and involved with multiple cytokines and metabolic pathways (38). It has been reported that cancer cells (HeLa, A549, and Myc-Cap/CR) treated with IL-17A induced more M2 polarization of RAW264.7 and THP-1 cells, which may be a result of the cyclooxygenase 2/prostaglandin E_2_ (COX-2/PGE_2_) pathway in the cancer cells (39). In addition, IL-17 was reported to directly induce THP-1-derived macrophages and mouse peritoneal macrophages into M2-like phenotypes via NF-κB activation (40). In our study, IL-17 or OC cells directly induced more M1-like macrophage phenotypes, respectively. However, when OC cells were pretreated with IL-17, M1-like phenotypes induced by OC cells alone polarized into more M2-like phenotypes. Thus, the coexistence of OC cells and IL-17A may produce synergistic benefits to induce M2 polarization of macrophages.

Despite some valuable phenomena that have been discovered in this work, limitations should not be ignored. First, there was no statistically significant difference in tumor size between C. albicans infected and uninfected C3H mice, which may be due to the limited number of mice. Second, this study mainly focused on the IL-17A/IL-17RA-macrophage axis but ignored other immune cell subtypes in C. albicans promoting OC. As we have discovered, the CD4^+^ T cells and B cells were downregulated, but the MDSCs in peripheral blood were upregulated when 4NQO mice were infected with C. albicans. Especially, MDSCs are accepted as T-cell immunosuppressive cells (41). Thus, our further work will explore the role of T cells and MDSCs in C. albicans promoting OC. Finally, more clinical evidence is needed to demonstrate the correlations between C. albicans, IL-17A/IL-17-RA, macrophage, and cancer.

In summary, we demonstrated that C. albicans infection promoted OC incidence and progression in mice models. Further data found that OC cells with IL-17A/IL-17RA activation during C. albicans infection attracted macrophages into TME. Meanwhile, the attracted macrophages polarized into M2-like TAMs with PD-L1 and GAL-9 upregulation, which finally induced an immunosuppressive microenvironment to promote OC development. Thus, the treatment of C. albicans infection in oral potentially malignant disorders and oral cancer patients will be beneficial in delaying cancer development.

## MATERIALS AND METHODS

### Mice.

All animal procedures described in this study were reviewed and approved by the Biomedical Ethics Committee of Peking University (LA2021388). C57BL/6N male mice (6 to 8 weeks) and C3H/HeN male mice (6 to 8 weeks) were used. All mice were maintained under specific pathogen-free conditions and in the same facility and housing unit.

### Cell lines and culture conditions.

Mouse oral squamous cell carcinoma cell line (SCC VII) and macrophages (RAW 264.7) were used. SCC VII and RAW 264.7 cells were cultured in high-glucose Dulbecco’s modified Eagle’s medium (DMEM) (HyClone) supplemented with 10% fetal bovine serum (FBS) (HyClone) and 100 U/mL penicillin-streptomycin (Invitrogen), respectively. Cells were maintained at 37°C in the presence of 5% CO_2_.

### C. albicans and culture conditions.

C. albicans strain SC5314 was grown at 37°C in yeast extract peptone dextrose (YPD) medium (Solarbio, CN) overnight.

### The 4NQO-induced mouse tongue carcinogenesis model.

C57BL/6N mice were randomly divided into three groups (*n* = 6 per group): control group (4NQO), C. albicans infection group (4NQO + CA), and antifungal group (4NQO + CA + FCZ). From week 0, all three groups were fed with carcinogen 4NQO (Sigma) in the drinking water (100 μg/mL), which lasted for 16 weeks. From week 4, the mice in the 4NQO + CA group and 4NQO + CA + FCZ group were infected with C. albicans on the dorsum of the tongue by a cotton swab soaked with 200 μL of C. albicans suspension (6 × 10^8^ CFU/mL), while the mice in the 4NQO group were applied with medium alone, once every 3 days. From week 16, 4NQO water was removed. The mice in the 4NQO group and the 4NQO + CA group were fed with normal water, while the mice in the 4NQO + CA + FCZ group were fed with 0.5 mg/mL fluconazole (FCZ; Sigma) in drinking water and lasted for 4 weeks (Fig. 1A).

### The tongue tumor-bearing mouse model (C3H/HeN-SCC VII).

C3H/HeN mice were used for the tongue tumor-bearing mouse model. From week −1, the mice were fed with 0.1% (wt/vol) tetracycline hydrochloride (TCN; Sigma) in drinking water for 1 week to suppress the endogenous microflora (18). From week 0, the mice in C. albicans-infected group (CA) were infected with C. albicans on the dorsum of the tongue by a cotton swab soaked with 200 μL of C. albicans suspension (6 × 10^8^ CFU/mL, once every 3 days, 2 weeks); meanwhile, these mice were treated with 10^5^ CFU/mL C. albicans in drinking water (changed daily to prevent C. albicans from settling) until the sacrifice. The mice in the uninfected group (CON) were treated with medium alone on the dorsum of the tongue (once every 3 days, 2 weeks) and fed with normal water. After 2 weeks of preinfection, all of the mice were injected with SCC VII cells (2.5 × 10^5^ cells in a 25-μL volume) into the submucosa of the tongue dorsum and lasted for 2 weeks. The tumor volume was calculated using the formula: 1/2 × length (mm) × [width (mm)]^2^.

### Tumor RNA extraction, sequencing, and transcriptome analysis.

RNA-seq assay was performed by Shanghai Biotechnology Corporation. Briefly, total RNA was extracted from the tumor of the mice in the 4NQO, 4NQO + CA, and 4NQO + CA + FCZ groups by the use of miRNeasy minikit (Qiagen, GmBH, Germany) and purified by RNAClean XP kit (Beckman Coulter, Inc. Kraemer Boulevard Brea, CA, USA) and RNase-Free DNase Set (Qiagen, GmBH, Germany). cDNA library was constructed using VAHTS Universal V6 RNA-seq Library Prep kit for Illumina (Vazyme, USA) and then sequenced with Illumina NovaSeq6000. The sequencing reads were genome mapped using Hisat2 (version: 2.0.4). Stringtie (version: 1.3.0) was used to calculate reads per kilobase per million mapped reads (RPKM) values. The edgeR was used to analyze the differentially expressed genes with the following filter criteria: *q* value of ≤0.05 and fold change of ≥2.

### *In vivo* IL-17A neutralization and depletion of macrophages.

For IL-17A neutralizing experiments, C3H/HeN-SCC VII mice were injected both intraperitoneally (50 μg, 2 times/week) and locally (25 μg, 3 times/week) with anti-mouse IL-17A antibody (clone 17F3, BioXcell) for 2 weeks. For depletion of macrophages, C3H/HeN-SCC VII mice were injected locally with clodronate liposomes (25 μL, 3 times/week; Liposoma BV, The Netherlands) (42) for 2 weeks.

### Transwell migration experiments.

Transwell chamber (8.0-μm pore membranes; Corning) was used to explore the migration of macrophages. SCC VII cells (1 × 10^4^ cells per well) were seeded in the lower chamber and incubated for 24 h. Then, the culture medium was replaced with 600 μL serum-free medium supplemented with 0, 50, or 100 ng/mL recombinant mouse IL-17A (rmIL-17A, 421-ML, R&D Systems) and cultured for 8 h. After that, 200 μL RAW 264.7 cells (5 × 10^5^/mL) in serum-free medium were added into the upper chamber. After being cocultured for 24 h, the RAW 264.7 cells remaining on the upper surface of the membrane were removed, and the cells passed onto the lower surface of the membrane were fixed, stained with 0.1% crystal violet solution, and numbered under the microscope.

### Coculture experiments.

To explore the influence of OC cells stimulated with IL-17A on macrophages, RAW 264.7 cells were cultured in culture supernatants from SCC VII cells treated with 0 or 100 ng/mL rmIL-17A for 8 h. After being cultured for 24 h, the RAW 264.7 cells were collected for RT-qPCR and FCM.

### Immune cell phenotyping by flow cytometry.

Peripheral blood mononuclear cells were separated by density gradient centrifugation. Tumor-infiltrating immune cells were separated by mechanically homogenized and filtered with a 70-μm cell strainer, followed by CD45^+^ staining and gating. The antibodies for leukocyte subpopulations are listed in Table S2. The stained cells were analyzed using a flow cytometer (CytoFlEX S, Beckman Coulter). FlowJo software (v. 10) was used for data analysis.

10.1128/mbio.00447-23.7 TABLE S2 Antibodies used in this study. Download Table S2, DOCX file, 0.02 MB. Copyright © 2023 Wang et al. 2023 Wang et al. https://creativecommons.org/licenses/by/4.0/ This content is distributed under the terms of the Creative Commons Attribution 4.0 International license.

### Quantitative real-time PCR.

Total RNA was extracted with TRIzol Reagent (Invitrogen) and reverse-transcribed into cDNA with ABScript III Reverse Transcriptase (Abclonal, Wuhan, China). Then, the amplification was performed in triplicate using Universal SYBR green Fast qPCR Mix (Abclonal, Wuhan, China). The melting curves were checked and the relative gene expression was analyzed using the 2^−ΔΔCt^ method with normalization to GAPDH and ACTB. The primer (Sangon Biotech, Shanghai, China) sequences are listed in Table S3.

10.1128/mbio.00447-23.8 TABLE S3 Primers for quantitative RT-PCR analysis. Download Table S3, DOCX file, 0.02 MB. Copyright © 2023 Wang et al. 2023 Wang et al. https://creativecommons.org/licenses/by/4.0/ This content is distributed under the terms of the Creative Commons Attribution 4.0 International license.

### Hematoxylin and eosin, immunohistochemistry, and immunofluorescence.

After formaldehyde fixation, dehydration, and paraffin embedding, the tissues were cut into 5 μm sections and stained with hematoxylin and eosin (H&E), IL-17RA, F4/80, CD86, CD163, CCL2, GAL-9, and PD-L1 (catalog number and manufacturer for antibodies are listed in Table S2). Images were taken using a fluorescence microscope (Olympus Co.) or a confocal laser scanning microscopy (TCS-SP8 DIVE; Leica).

### Enzyme-linked immunosorbent assay.

Enzyme-linked immunosorbent assay (ELISA) method was performed according to the instruction manual to detect the concentration of S100A9 and CCL2 in culture supernatants of SCC VII cells and RAW 264.7 cells as well as the tissue homogenization of tumor.

### Western blot.

Total proteins in cells and tissues were extracted by radioimmunoprecipitation (RIPA)-buffer (Thermo Fisher Scientific) added with protease and phosphatase inhibitors (Solarbio, CN). The Western blot (WB) experiment was performed according to the standard procedures. Primary antibodies against CCL2 were used at a 1:1,000 dilution.

### Statistics.

Each experiment was repeated three times unless otherwise stated. GraphPad Prism (v.8.0) was used for statistical analyses. Data are presented as mean ± standard deviation (SD). Data were statistically assessed for normality and variance homogeneity using the Shapiro-Wilk test and *F* test, respectively. Student’s two-tailed *t* test was used to determine the statistical relevance between the two groups. Pearson’s correlation was used to analyze the correlation between macrophage infiltration level and IL-17A/IL-17RA signature genes. A *P* value of <0.05 was indicative of statistical significance (*, *P* < 0.05; **, *P* < 0.01; ***, *P* < 0.001; ****, *P* < 0.0001).

### Data availability.

Sequence data that support the findings of this study have been deposited in NCBI BioProject with the primary accession code PRJNA944176 and in NCBI Sequence Read Archive (SRA) with the primary accession code SRP427315.
