# Supplementary figures and images for: Candida albicans Promotes Oral Cancer via IL-17A/IL-17RA-Macrophage Axis
Source: mBio. 2023 Apr 17;14(3):e00447-23. doi: 10.1128/mbio.00447-23 (PMC10294694; doi:10.1128/mbio.00447-23)

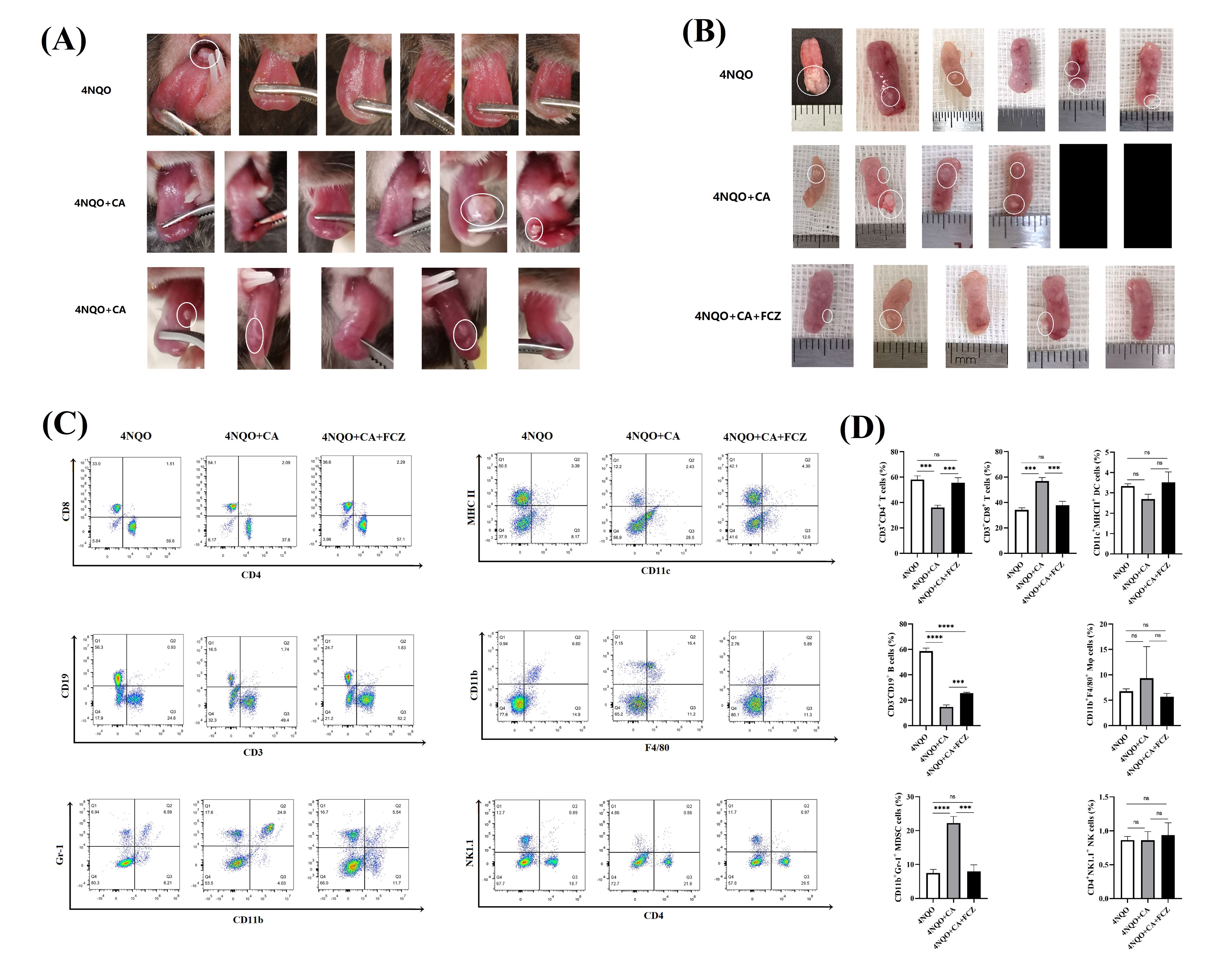

Supplement: FIG S1 [file mbio.00447-23-s0004.tif]

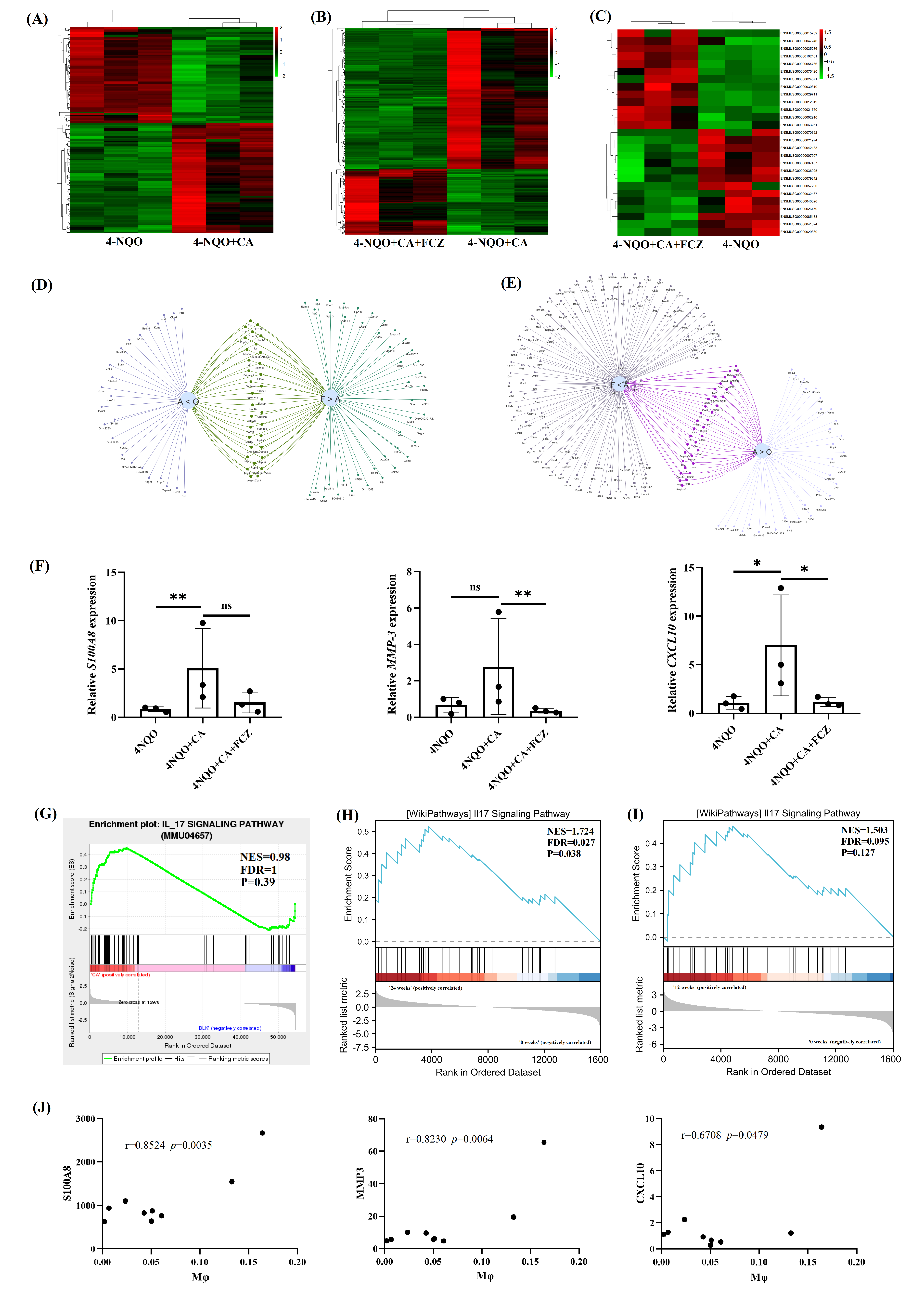

Supplement: FIG S2 [file mbio.00447-23-s0005.tif]

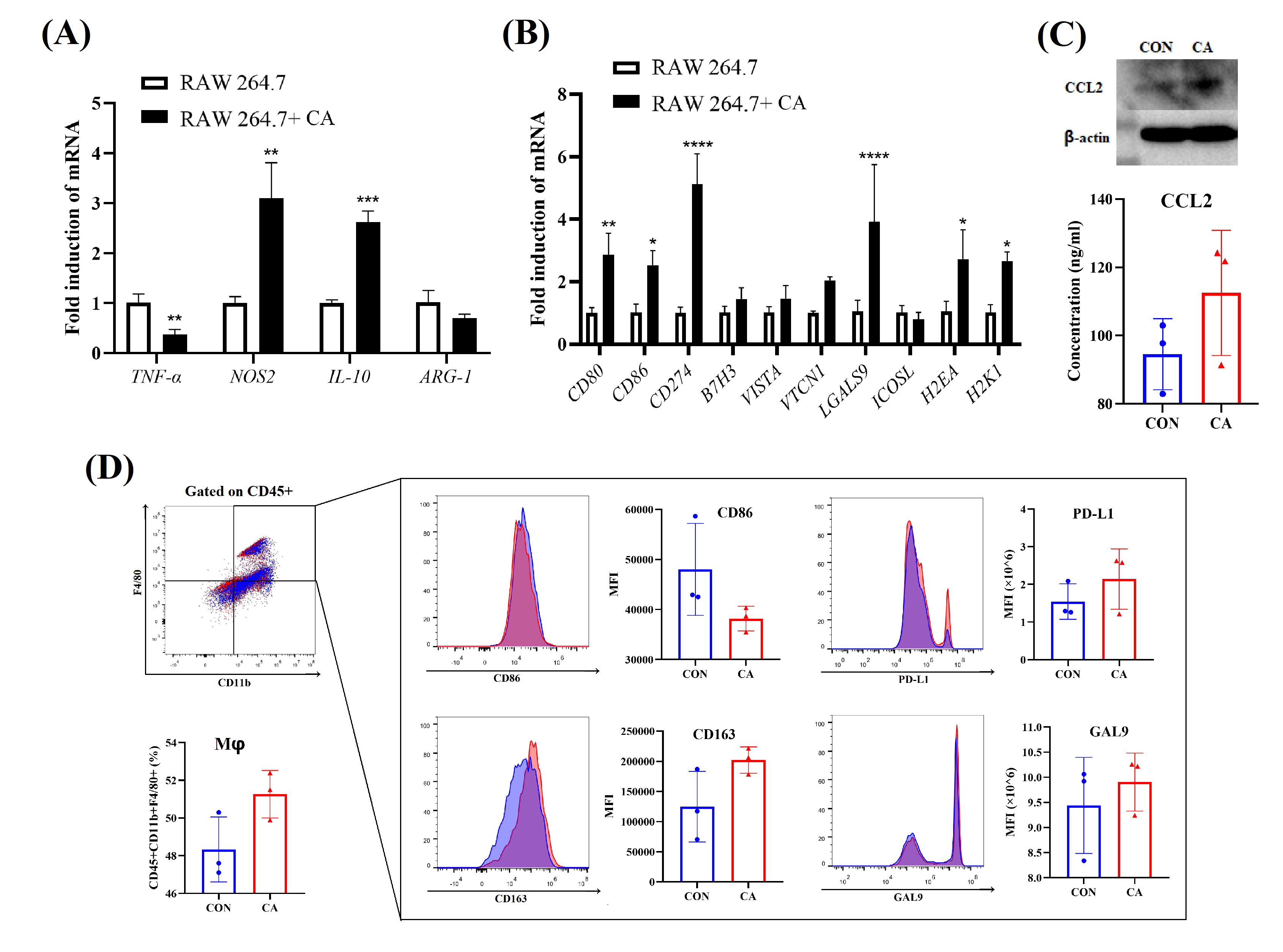

Supplement: FIG S3 [file mbio.00447-23-s0006.tif]

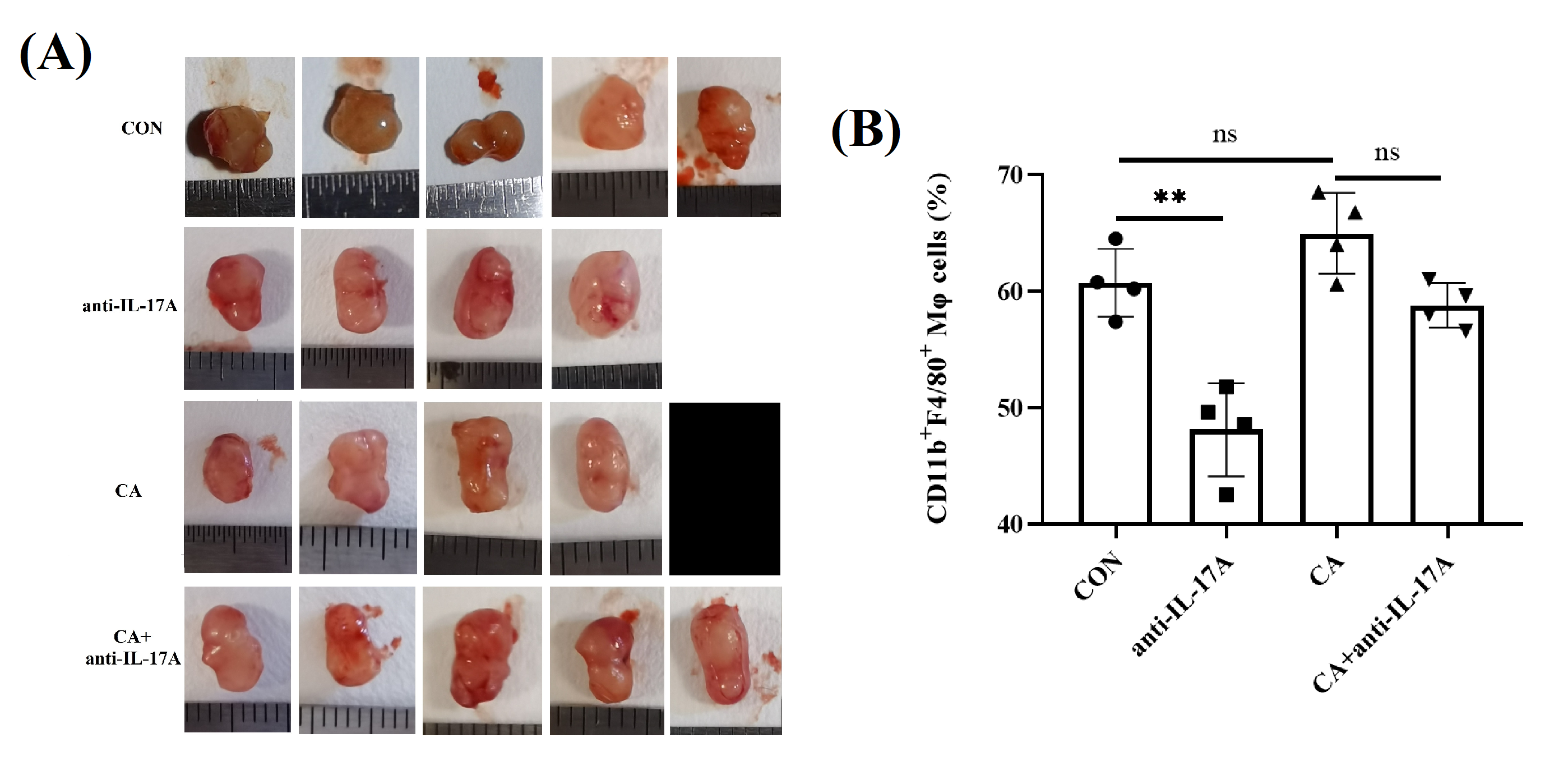

Supplement: FIG S4 [file mbio.00447-23-s0007.tif]

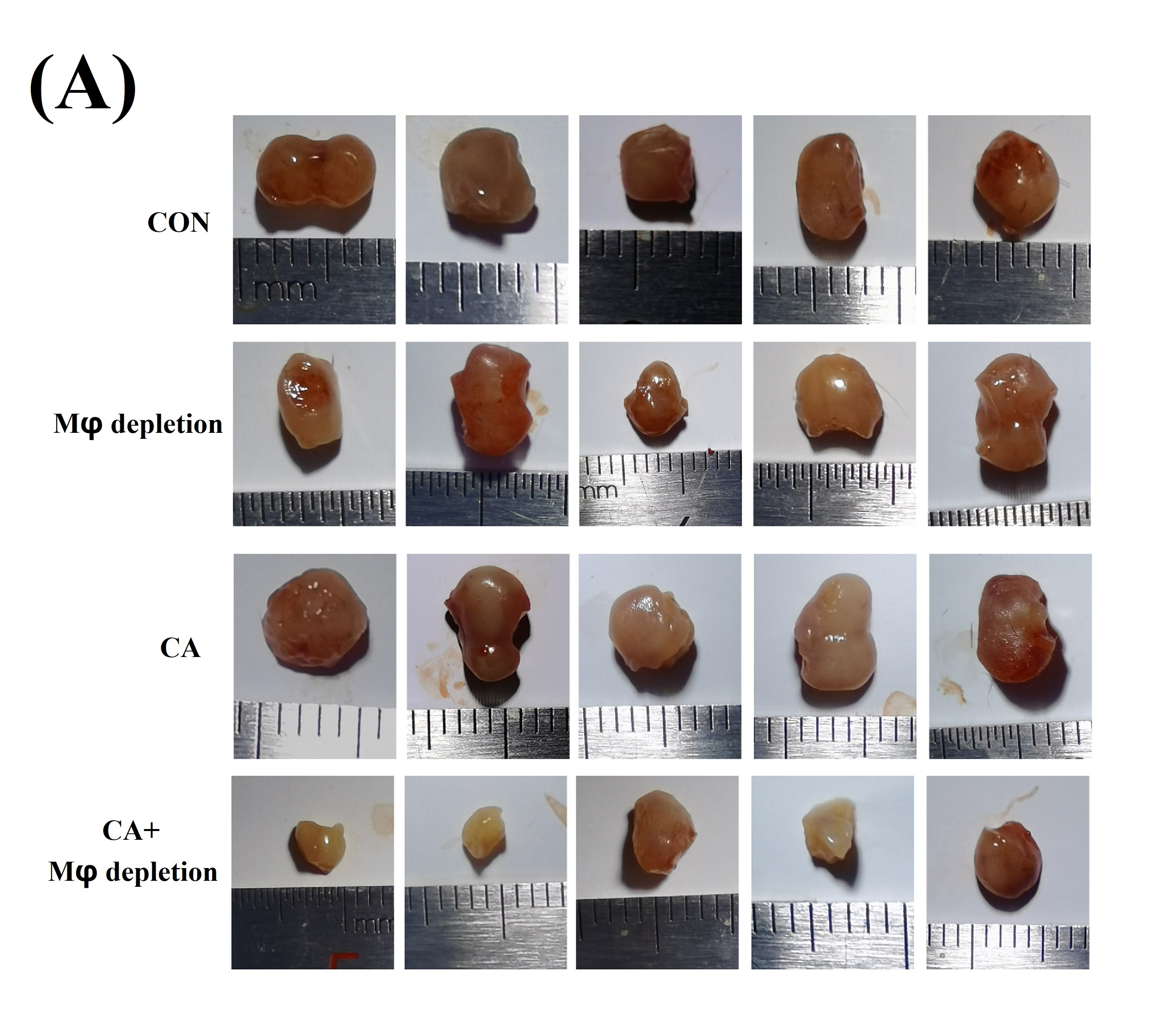

Supplement: FIG S5 [file mbio.00447-23-s0008.tif]
