# Supplementary material for: Candida albicans Promotes Oral Cancer via IL-17A/IL-17RA-Macrophage Axis
Source: mBio. 2023 Apr 17;14(3):e00447-23. doi: 10.1128/mbio.00447-23 (PMC10294694; doi:10.1128/mbio.00447-23)
Supplement: TABLE S2 [file mbio.00447-23-s0002.docx]

**Table S2.** Antibodies used in this study

| Antibody | Cat# | Manufacturer |
| --- | --- | --- |
| APC/Fire^TM^ 750 anti-mouse CD45 | 103153 | BioLegend |
| FITC anti-mouse CD3 | 100203 | BioLegend |
| APC anti-mouse CD3 | 100235 | BioLegend |
| PE/Cyanine7 anti-mouse CD4 | 100421 | BioLegend |
| FITC anti-mouse CD4 | 100509 | BioLegend |
| APC anti-mouse CD8a | 100711 | BioLegend |
| PE anti-mouse CD8a | 100707 | BioLegend |
| APC anti-mouse CD11b | 101211 | BioLegend |
| PE/Cyanine7 anti-mouse CD11b | 101216 | BioLegend |
| PE anti-mouse CD19 | 152407 | BioLegend |
| PE anti-mouse F4/80 | 123109 | BioLegend |
| FITC anti-mouse F4/80 | 123107 | BioLegend |
| PE anti-mouse NK1.1 | 108707 | BioLegend |
| APC anti-mouse CD11c | 117309 | BioLegend |
| APC anti-mouse Gr-1 | 106411 | BioLegend |
| APC anti-mouse CD86 | 105012 | BioLegend |
| PE anti-mouse CD163 | 156704 | BioLegend |
| PE anti-mouse CD274 (PD-L1) | 124307 | BioLegend |
| PE anti-mouse Galectin-9 | 136103 | BioLegend |
| Anti-CCL2 | DF7577 | Affinity Biosciences |
| Anti-F4/80 | ab240946 | abcam |
| Anti-CD274 (PD-L1) | ab213480 | abcam |
| Anti-Galectin-9 | AF5290 | Affinity Biosciences |
| CD86 | #19589 | Cell Signaling Technology |
| CD163 | DF8235 | Affinity Biosciences |
| IL-17RA | DF3602 | Affinity Biosciences |
| Ki-67 | ab16667 | abcam |
| InVivoMAb anti-mouse IL-17A | BE0173 | Bio Xcell |
| clodronate liposomes | CP-005-005 | Liposoma BV |
