# Supplementary material for: Candida albicans Promotes Oral Cancer via IL-17A/IL-17RA-Macrophage Axis
Source: mBio. 2023 Apr 17;14(3):e00447-23. doi: 10.1128/mbio.00447-23 (PMC10294694; doi:10.1128/mbio.00447-23)
Supplement: TABLE S3 [file mbio.00447-23-s0003.docx]

**Table S3.** Primers for quantitative RT-PCR analysis

| Gene | Forward primer (5’-3’) | Reverse primer (5’-3’) |
| --- | --- | --- |
| *S100A9* | ACCACCATCATCGACACCTTC | AAAGGTTGCCAACTGTGCTTC |
| *S100A8* | GACAATGCCGTCTGAACTGG | GCTACTCCTTGTGGCTGTCTT |
| *MMP-3* | GGCCTGGAACAGTCTTGGC | TGTCCATCGTTCATCATCGTCA |
| *CXCL10* | CCAAGTGCTGCCGTCATTTTC | GGCTCGCAGGGATGATTTCAA |
| *CSF1* | ATGAGCAGGAGTATTGCCAAGG | TCCATTCCCAATCATGTGGCTA |
| *CCL2* | TTAAAAACCTGGATCGGAACCAA | GCATTAGCTTCAGATTTACGGGT |
| *CCL3* | TTCTCTGTACCATGACACTCTGC | CGTGGAATCTTCCGGCTGTAG |
| *CCL4* | TTCCTGCTGTTTCTCTTACACCT | CTGTCTGCCTCTTTTGGTCAG |
| *CCL5* | GCTGCTTTGCCTACCTCTCC | TCGAGTGACAAACACGACTGC |
| *ARG-1* | CATATCTGCCAAAGACATCGTG | GACATCAAAGCTCAGGTGAATC |
| *IL-10* | GCTCTTACTGACTGGCATGAG | CGCAGCTCTAGGAGCATGTG |
| *NOS2* | GTTCTCAGCCCAACAATACAAGA | GTGGACGGGTCGATGTCAC |
| *TNF-α* | CCCCAAAGGGATGAGAAGTT | CACTTGGTGGTTTGCTACGA |
| *CD80* | ACCCCCAACATAACTGAGTCT | TTCCAACCAAGAGAAGCGAGG |
| *CD86* | TGTTTCCGTGGAGACGCAAG | TTGAGCCTTTGTAAATGGGCA |
| *CD274* | GCTCCAAAGGACTTGTACGTG | TGATCTGAAGGGCAGCATTTC |
| *B7H3* | ATGCTTCGAGGATGGGGTG | CCAGGCTCTGGGGAAAAGG |
| *VISTA* | CAACACCCAAGGAATCGAAAAC | ATCTAGGGATGGGAAAAAGACG |
| *VTCN1* | CTTTGGCATTTCAGGCAAGCA | TGATGTCAGGTTCAAAAGTGCAG |
| *LGALS9* | ATGCCCTTTGAGCTTTGCTTC | AACTGGACTGGCTGAGAGAAC |
| *ICOSL* | TAAAGTGTCCCTGTTTTGTGTCC | ATTGCACCGACTTCAGTCTCT |
| *H2K1* | ACCAGCAGTACGCCTACGA | AACCAGAACAGCAACGGTCG |
| *H2EA* | AAGTCATGGGCTATCAAAGAGGA | CTCATCGCCGTCAAAGTCAAA |
| *ACTB*  *GAPDH* | Cat#B661302-0001, Sangon Biotech, Shanghai  Cat#B661304-0001, Sangon Biotech, Shanghai | |
